# Imiquimod-Loaded Chitosan-Decorated Di-Block and Tri-Block Polymeric Nanoparticles Loaded In Situ Gel for the Management of Cervical Cancer

**DOI:** 10.3390/gels9090713

**Published:** 2023-09-03

**Authors:** Aliyah Almomen, Mohamed Badran, Adel Ali Alhowyan, Musaed Alkholief, Aws Alshamsan

**Affiliations:** 1 Department of Pharmaceutical Chemistry, College of Pharmacy, King Saud University, Riyadh 11495, Saudi Arabia; 2 Department of Pharmaceutics, College of Pharmacy, King Saud University, Riyadh 11495, Saudi Arabia; mbadran@ksu.edu.sa (M.B.); adel-ali@ksu.edu.sa (A.A.A.); malkholief@ksu.edu.sa (M.A.); aalshamsan@ksu.edu.sa (A.A.)

**Keywords:** imiquimod, vaginal drug delivery, nanoparticles, chitosan, in situ gels, cervical cancer

## Abstract

Background: Cervical intraepithelial neoplasia, the predisposing factor for cervical cancer (CC), is caused by human papillomavirus (HPV) infection and can be treated with imiquimod (IMQ). However, poor water solubility and side effects such as local inflammation can render IMQ ineffective. The aim of this study is to design a prolonged release nano system in combination with mucoadhesive–thermosensitive properties for an effective vaginal drug delivery. Methods: Polylactic-co-glycolic acid (PLGA), polycaprolactone (PCL), poly lactide-co-caprolactone (PLA-PCL), and poly L-lactide-co-caprolactone-co-glycolide (PLGA-PCL) were used to create IMQ nanoparticles. Chitosan (CS) was then added to the surfaces of the IMQ NPs for its mucoadhesive properties. The NPs were then incorporated into poloxamer hydrogels. The NPs’ size and morphology, encapsulation efficiency (EE), in vitro drug release, gel characterization, ex vivo drug permeation, and in vitro safety and efficacy were characterized. Results: Two batches of NPs were prepared, IMQ NPs and CS-coated NPs (CS-IMQ NPs). In general, both types of NPs were uniformly spherical in shape with average particle sizes of 237.3 ± 4.7 and 278.2 ± 5.4 nm and EE% of 61.48 ± 5.19% and 37.73 ± 2.88 for IMQ NPs and CS-IMQ NPs, respectively. Both systems showed prolonged drug release of about 80 and 70% for IMQ NPs and CS-IMQ NPs, respectively, within 48 h. The gelation temperatures for the IMQ NPs and CS-IMQ NPs were 30 and 32 °C, respectively; thus, suitable for vaginal application. Although ex vivo permeability showed that CS-IMQ NPs showed superior penetration compared to IMQ NPs, both systems enhanced drug penetration (283 and 462 µg/cm^2^ for IMQ NPs and CS-IMQ NPs, respectively) relative to the control (60 µg/cm^2^). Both systems reduced the viability of cervical cancer cells, with a minimal effect of the normal vaginal epithelium. However, IMQ NPs exhibited a more pronounced cytotoxic effect. Both systems were able to reduce the production of inflammatory cytokines by at least 25% in comparison to free IMQ. Conclusion: IMQ and CS-IMQ NP in situ gels enhanced stability and drug release, and improved IMQ penetration through the vaginal tissues. Additionally, the new systems were able to increase the cytotoxic effect of IMQ against CC cells with a reduction in inflammatory responses. Thus, we believe that these systems could be a good alternative to commercial IMQ systems for the management of CC.

## 1. Introduction

Cervical intraepithelial neoplasia (CIN) is a condition that precedes cervical cancer (CC), the second most common cancer in women worldwide causing over 270,000 deaths annually [1]. The primary causes of CIN and invasive CC are the human papillomavirus (HPV) virus strains 16 and 18. Current treatments for high-grade CIN involves surgical excision such as loop electrosurgical excision [2]. However, this mode of treatment is associated with the recurrence rate and the possible development of CIN grade 2 or higher within 5 to 10 years [2]. Furthermore, it was reported that patients who underwent surgery had a longer time to conception compared to those in the control group [3]. Moreover, pregnancies within a short time interval of having surgical excision of CIN have been linked to an increased risk of spontaneous abortion [2]. Therefore, non-surgical alternatives for managing CIN are needed. 

Imiquimod (IMQ) is an immune modulator that exhibits antiviral and antitumor activity. It induces the expression of cytokines, such as interferon, tumor necrosis factor, and interleukins 1, 6, and 8, and activates T-cells, resulting in a tumor-directed immune response associated with the clearance of HPV [3]. IMQ has been approved for the management of genital warts caused mostly by HPV types 6 and 11. A study showed that IMQ is effective in treating CIN associated with HPV infection [4]. In fact, IMQ has been shown to lead to the complete remission of high-grade CIN, which is significantly higher compared to control patients [5]. However, IMQ’s poor water solubility can affect its bioavailability. Furthermore, IMQ local and systemic side effects such as burning, pain, tenderness, vesicular eruptions, and ulcerations may limit its effectiveness, necessitating the use of a drug delivery system [6]. 

Polymeric nanoparticles (NPs) are being actively researched for their targeted drug delivery due to them providing many advantages such as the sustained release of a drug at the target site and the improved local availability, which can enhance therapeutic efficacy and reduce adverse effects [7]. Researchers have explored copolymerizing and multiple approved polymers to create materials with a wide range of physical, mechanical, and biopharmaceutical properties [8]. Polylactic acids (PLAs) have gained significant attention in drug delivery and targeted applications over the past two decades [9]. PLAs are biodegradable, making them favorable for biomedical applications that can be eliminated safely [10]. Moreover, polylactic-co-glycolic acid (PLGA) NPs have been widely used for the targeted delivery of various anticancer drugs [11]. However, the hydrophobic properties of PLGA and the slow drug release rates of incorporated drugs are significant obstacles to forming efficient NPs [12]. Another biodegradable polymer that has found use in several drug delivery systems is polycaprolactone (PCL) [13]. PCL is easily shaped into various forms, enabling a variety of biomedical applications [14]. Thus, di-block and tri-block copolymers of PLA-PCL and PLGA-PCL have been proposed to address the limitations of individual polymers in biomedical applications [15,16]. PLGA, PCL, and their copolymers are safe and effective drug delivery systems.

Mucoadhesive polymeric systems such as chitosan (CS) have previously been used to improve the residence time of vaginal drug delivery systems [15,16]. CS binds to mucin through various mechanisms such as hydrogen bonds, electrostatic interactions, polymer chain interdiffusion and van der Waal forces [16]. When used to coat polymeric NPs, CS was shown to be able to enhance the mucus surface retention time and reduce the loss of NPs because of vaginal secretion [15,16]. 

Poloxamer in situ gel-forming systems have gained significant attention in the field of drug delivery due to their ability to transform from a liquid formulation into a gel-like state at the target site [15]. Moreover, poloxamer can exhibit some degree of mucoadhesion when applied to mucosal surfaces [15]. The combination of CS NPs and in situ gel systems can offer a versatile platform for localized drug delivery. Therefore, this study aims to enhance the efficacy and reduce the side effects associated with IMQ by designing a local vaginal drug delivery system through the loading of IMQ into NPs coated with CS and loaded in an in situ hydrogel. 

## 2. Results and Discussion

### 2.1. Particle Size and Zeta Potential

The type of polymer selected has a big impact on how well NPs form. In this study, various polymers and copolymers were tested to ensure that IMQ was effectively encapsulated. The resulting NPs showed nanometer-sized particle sizes, demonstrating successful formation and the right size distribution. Table 1 lists the results of the zeta potential, polydispersity index, and particle size measurements for each of the prepared formulations. The average particle sizes of the IMQ NPs were as follows: 219.4 ± 8.1 nm for F1, 189.1 ± 3.9 nm for F2, 227.3 ± 4.7 nm for F3, and 206.7 ± 8.7 nm for F4 (Table 1). Among these formulations, PLA-PCL produced the largest particle size compared to other IMQ NPs. On the other hand, the smallest particle size was observed in PCL, and it was significantly smaller than PLGA. The smaller particle size achieved with PCL compared to PLGA can be attributed to the enhanced flexibility of the PCL chain [17]. Additionally, the lower glass transition temperature of PCL allows for greater polymer chain mobility during the process, resulting in the formation of smaller nanoparticles compared to PLGA [18].

It is clear from comparing the particle sizes of the formulations that there is a strong correlation between the mean molecular weight of a polymer and the size of the prepared NPs. The large particle size was confirmed by increasing the polymer’s molecular weight [19]. The large particle size of IMQ NPs may be caused by the formation of highly viscous dispersions [20]. Additionally, the potential for hydrogen bonding between the carboxylic groups of PLGA and the carbonyl groups of PCL (F4) may lead to a reduction in intermolecular spaces and smaller particle sizes [21]. 

To increase the antitumor properties, IMQ NP formulations were coated with CS for high cellular uptake [22]. According to the copolymer used and the CS coating, the particle sizes of CS-IMQ NPs significantly increased, as shown in Table 2. The mean particle sizes for CS-F1, CS-F2, CS-F3, and CS-F4, respectively, were 225.9, 232.4, 259.2, and 218.5 nm. The formation of a highly viscous suspension, which results in a larger particle size, could be attributed to the polymer used as well as the presence of the CS layer on the surface of IMQ NPs [23]. Their cellular uptake, where the internalization processes take place sequentially, depends significantly on the particle size and surface charge. It is crucial to consider the surface charge of NPs when determining in vivo cell membrane interaction because it may significantly affect their stability through strong electrostatic attraction [24]. Additionally, all varieties of NPs displayed low PDIs, indicating a system that is highly monodispersed, which is strongly advised in nanomedicine. 

Due to the presence of carboxyl-terminal groups on PLGA molecules on the surface of F1 and F4 molecules, the zeta potential of uncoated IMQ NPs was negative and ranged from −13.63 mV to −20.13 mV [25]. The uncoated polymeric NPs had a zeta potential in the range of −14 to −20 mV, which may be related to the presence of carboxyl groups on the NPs’ surfaces [26]. After CS addition, all samples clearly showed a positive zeta potential due to the amine groups in the CS structure. This observation confirmed the successful CS coating onto the surfaces of polymeric NPs through physical means. It has been reported that when the ratio of CS to polymeric NPs is low, the zeta potential of CS NPs formed by physical adsorption is larger than that of those prepared via chemical binding [25]. Thus, physical adsorption is preferable to chemical binding when the concentration of CS is low. The ability of the positively charged NPs to internalize the cell membrane could increase their uptake by cells [24]. Positive zeta potential values were present in all of the prepared CS NPs. The findings suggest that the amino groups on the CS molecule are located on the surface of the NPs because the zeta potentials of the CS-IMQ NPs were found to have been shifted to positive values. These outcomes clearly demonstrate the successful CS coating of the IMQ NPs’ surface. Additionally, CS-coated IMQ NPs are positively charged. In addition, positively charged CS-coated IMQ NPs can interact with the negatively charged cell membrane through ionic interactions, which could contribute to the increase in residence time [25]. The adsorption of CS onto the surface of polymeric NPs involves interactions such as hydrogen bonding and electrostatic interactions [25,26].

### 2.2. Encapsulation Efficiency and Loading Capacity 

The amount of encapsulated IMQ in the different NPs was evaluated based on the polymers and copolymers used. Table 2 displays the results of EE% and DL%. The composition of the formulation plays a main role in the loading of IMQ into NPs. In comparison to their single polymers, the copolymers in the preparation of IMQ NPs were shown to have a reasonable EE% and LC%. Hence, the EE% and LC% for both CS-F4 and F4 formulations were relatively high compared to the other formulations as indicated in Table 1. 

The yield% was relatively high in all formulations ranging from 73% to 86%, with the highest values observed for NPs based on PLGA-PCL copolymers. The EE% values of F1, F2, F3, and F4 were 51, 47, 40, and 61.5%, respectively, while LC% values ranged between 6.4 and 10.3%, respectively. The highest value of EE% for F4 could be attributed to the presence of different polymers, which together create a polymer network characterized by an irregular and disordered crystalline structure [13]. This unique structure is believed to facilitate the accommodation of IMQ within the network. On the other hand, the lowest EE% value in the case of F3 copolymer can be explained by the partial and rapid precipitation of the polymer that occurs after the addition of the organic phase to the surfactant solution [13].

Table 2 presents the impact of CS coating on the efficacy of IMQ NPs. The results show that the EE% values for CS-F1, CS-F2, CS-F3, and CS-F4 were 46%, 39%, 37.7%, and 49.3%, respectively, while the drug loading capacity (LC%) ranged between 5.4% and 7.7% (Table 2). The decrease in EE% and LC% in CS-coated IMQ NPs can be attributed to the loss of IMQ during the coating process, particularly the molecules present on the surface of the NPs. Subsequently, LC% was decreased due to the increase in the amount of the polymer in the NPs with respect to the fixed amount of drug loading [. The decrease in EE% observed with CS NPs is consistent with the results of previous studies examining formulations with CS coating [26,27]. Thus, the possible reduction in EE% should be considered when CS coating is intended with NP-based drug delivery systems. 

### 2.3. In Vitro Release Studies 

**Figure 1:**
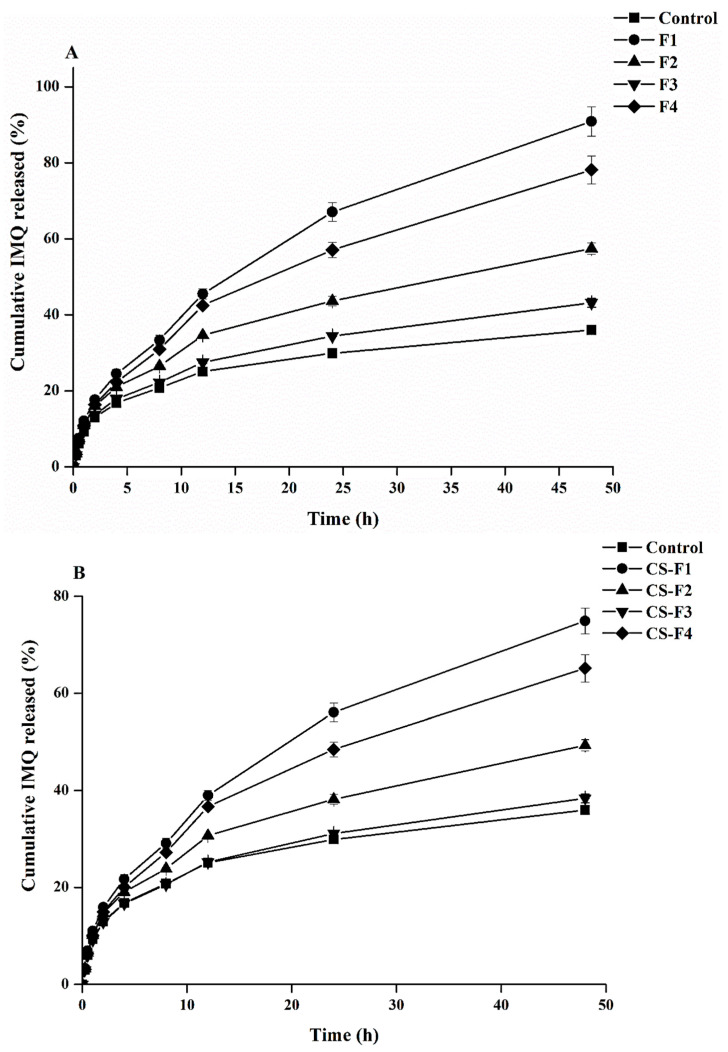
In vitro drug release patterns of IMQ NPs (**A**) and CS-IMQ NPs (**B**).

The in vitro drug release profiles of IMQ NPs and CS-IMQ NPs are presented in Figure 1A,B, respectively. The NP formulations displayed a large initial burst release. At 4 h, the burst release rates of F1, F2, F3, and NPs-F4 were observed to be 24%, 21%, 18%, and 22%, respectively. The burst effect is caused by some of the IMQ that was adsorbing to the NPs being readily released by diffusion. Additionally, the smaller size may have increased the surface area, increasing the release as in NPs-F1 and F2 [27]. However, after 48 h, 91%, 57%, 43%, and 78% of IMQ was released from F1, F2, F3, and F4, respectively. According to Wang et al., slow release was caused by polymer swelling, pore diffusion, followed by polymer erosion, and degradation [28]. Overall, F3 and F2 exhibited the slowest drug release rates. On the other hand, the F1 (PLGA) and F4 (PLGA-PCL) formulations showed the highest burst release magnitudes. The employed copolymers significantly decreased the initial burst release while prolonging medication release. The slowest drug release rate observed with F2 (PCL formulation) may be a result of PLA’s and PCL’s high crystalline natures [29]. These polymers were consequently less permeable to water, which resulted in a very slow rate of breakdown [30]. The lactides’ impact on PCL’s crystallinity, which has been proven to decrease crystallinity, can be used to explain the difference release rate between F4 and F2 [29]. The observed quick release rate for both tri-block-based NPs (F3 and F4) can be interpreted in light of what Zhang et al. reported [31]. The high crystalline nature of PLA and PCL may be the reason for the F2 (PCL) formulation’s slowest drug release rate [29]. These polymers’ rates of breakdown were consequently very slow since they had less water permeability [30]. The F4 (PLGA-PCL) formulation and the F2 (PCL) formulation had different rates of release. The quick release rate observed for both tri-block-based NPs (F3 and F4) can be interpreted in light of the findings of Zhang et al. [31]. Drug release was significantly reduced in NPs composed of poly-L-lactic acid (PLA-PCL). About 22%, 19%, 17%, and 20% of 5-IMQ were released from CS-F1, CS-F2, CS-F3, and CS-F4, correspondingly, in the first 4 h. Additionally, the amounts of IMQ released from CS-F1, CS-F2, CS-F3, and CS-F4 were 75%, 49%, 38%, and 65%, respectively, after 48 h. Due to the opposing surface charge of CS that was applied to the surface of NPs during the surface coating procedure, the CS-surface-coated IMQ NPs displayed a lower burst release than the uncoated NPs. CS coating can form a diffusion barrier around the NPs, which helps control the release of the drug encapsulated within the NPs [32,33]. As a result, both IMQ NPs and CS-IMQ NPs showed moderate and sustained release kinetics. Since PLGA is more hydrophilic than PCL, more IMQ was released into the acetate buffer solution [11].

The in vitro release kinetic model of IMQ from the formulated systems was estimated using different mathematical models, comprising the zero-order, first-order, Higuchi, and Korsmeyer–Peppas kinetic models, as shown in Table 3. The correlation coefficient (R^2^) value was used to figure out the release kinetic model of IMQ, in which the mathematical model that has the highest R^2^ value is most likely to represent the release kinetic model. Accordingly, the data presented in Table 3, reveal that the release model of IMQ from obtained formulations fits with Korsmeyer–Peppas’ equation. As shown, the *n* value (release exponent) is used to characterize different release mechanisms. It was found that the *n* values were mostly less than 0.43, suggesting the release mechanism was governed by diffusion. This behavior implies that the drug released from the system follows a Fickian transport pattern. 

### 2.4. Surface Morphology

**Figure 2:**
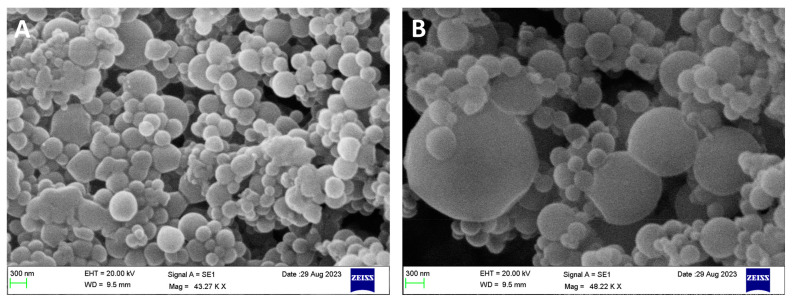
SEM images of (**A**) IMQ NPs (F4) and (**B**) CS-IMQ NPs (CS-F4).

SEM analysis demonstrated that the surface morphology of IMQ NPs (F4) (Figure 2A) and CS-IMQ NPs (CS-F4) (Figure 2B) appeared smooth and spherical in shape. However, it is important to note that due to the lyophilization process, the particles observed in SEM images may appear slightly larger than those detected by DLS measurements [27]. The presence of CS on the surface of IMQ NPs increased the adhesion between the particles due to the interaction between CS molecules. Furthermore, SEM provides an accurate measurement of the diameter of NPs in the dry state, while light scattering techniques are used to calculate the hydrodynamic diameter of NPs.

### 2.5. Characterization of the In Situ Hydrogels

These systems were successfully prepared, and their pH, gelation temperature, viscosity, and ex vivo vaginal permeation were all measured. In situ hydrogels had a pH between 4.5 and 5.4, which is in the same ballpark as vaginal fluids. Since the vagina is sensitive to pH, it is critical to regulate the formulation’s pH to prevent unpleasant side effects such as irritation.

#### 2.5.1. Determination of the Sol–Gel Transition Temperature 

This test was run to see if the formulations’ sol–gel transition temperatures were suitable for vaginal application. Hydrogels made from poloxamer were demonstrated to be transparent, clear, and colorless. The kind and quantity of poloxamer used had an impact on the hydrogels’ gelation temperature values. The sol–gel transition temperature for in situ hydrogels (P407 20%; P188) was found to be approximately 30 °C after the incorporation of F4. After the addition of CS-F4, the temperature was increased to 32 °C. The gelation time was 50 to 110 s. 

#### 2.5.2. Viscosity Measurement 

One of their preferred features is how in situ gel changes from a solution in the room temperature state to a gel when it interacts with biological circumstances. It is necessary to assess both their viscosity at a storage temperature and their physiological temperature. Here, the viscosity of the formulations was assessed at 25 and 34 °C. At 25 °C, the formulations, F4 and CS-F4, were in a liquid condition with respective viscosities of 734 and 922 cP. The formulations soon changed into a gel state and recorded 22,324 and 24,117 cP, respectively, when the environment’s temperature reached 34 °C. It is hoped that high-viscosity formulations will extend medication release and decrease treatment frequency. 

#### 2.5.3. Ex Vivo Permeability Study

IMQ NPs (F4) and CS-IMQ NPs (CS-F4) exhibited a high EE, a low particle size, and the controlled release of IMQ; therefore, they were selected for the ex vivo permeability study. 

**Figure 3:**
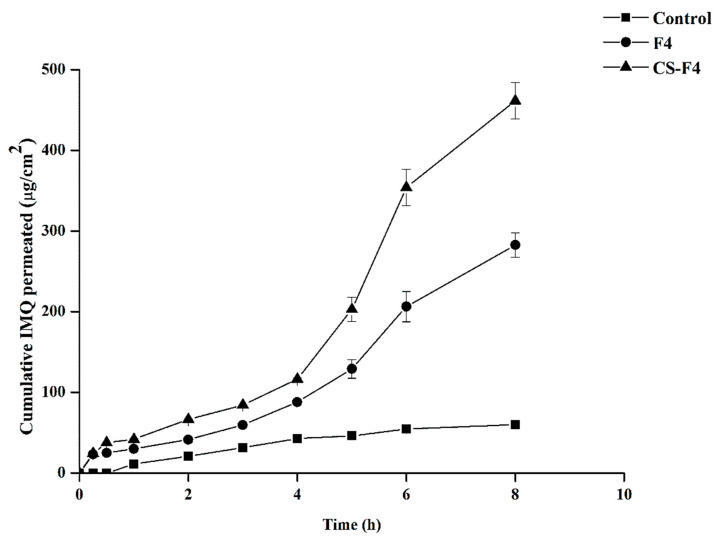
Ex vivo permeability profile of IMQ-loaded F4 and CS-F4 in situ gel formulations through the sheep vaginal mucosa at pH 4.5 citrate-phosphate buffer release medium at 37 °C.

The ex vivo permeability profile of the free IMQ suspension, F4, and the CS-F4 and IMQ permeation parameters are presented in Figure 3 and Table 4. The CS-coated formulation was chosen due to the mucoadhesive properties of CS and driven by its cationic character. This behavior enables ionic interactions between the cationic primary amino groups of CS and the anionic substructures of the mucus [32]. After 8 h, the control (pure IMQ), F4, and CS-F4 formulations achieved drug permeability values of 60, 283, and 462 µg/cm^2^, respectively. CS-F4 showed higher drug permeability compared to the uncoated one. The *Js* value of F4 was significantly increased (*p* < 0.05) compared to the pure IMQ suspension, and this value was extremely high (*p* < 0.05) for CS-F4. Similarly, the *p*-values for F4 and CS-F4 were significantly (*p* < 0.05) higher than that for the pure IMQ suspension. IMQ was enhanced by 21.5 and 29.8 times in IMQ-loaded F4 and CS-F4, respectively, compared to the control. These results are consistent with those reported by [27], who showed that CS-coated polymeric NPs had a significant effect on the targeted drugs. The findings above suggest that CS-coated PLGA-PCL NPs (CS-F4) exhibit better vaginal permeation parameters than PLGA-PCL NPs (F4), leading to a more sustained release of IMQ. It has been reported that polymeric NPs loaded in an in situ gel formulation for vaginal therapy of genital herpes observed a linear permeability graph in the ex vivo vaginal membrane permeability study up to 12 h [33]. 

### 2.6. In Vitro Cytotoxicity Studies

Imiquimod’s biological action includes the activation of TLR-7 and TLR-8, which in turn activate a signaling cascade that enhances nuclear factor kappa B (NF-B) translocation [34]. This results in the production of pro-inflammatory cytokines such as IFN-γ and TNF-α, and chemokines [34]. IMQ’s acute antiviral and anticancer effects are mostly attributable to its capacity to activate innate immune responses, which predominantly include the release of cytokines such as IFN-γ and IL-6 [34,35].

**Figure 4:**
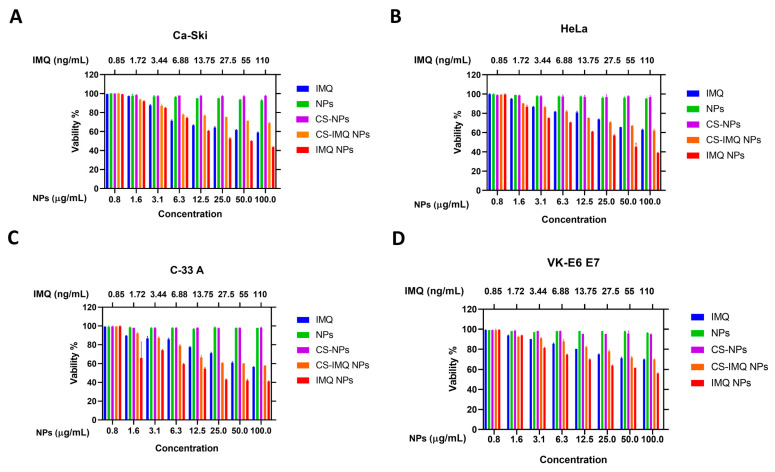
Cytotoxicity study in (**A**) Ca-Ski, (**B**) HeLa, (**C**) C-33 A, and (**D**) VK/E6 E7 cells after 72 h of treatment. Data are represented as mean ± SD (*n* = 3). Statistical significance was obtained with *p*-values ≤ 0.05. Statistical significance tables are available in the Supplementary Materials.

Our results showed that drug-free NPs exhibited no cytotoxicity to cancer cells or the normal vaginal epithelium in all concentration used. However, IMQ-loaded nanoparticles, i.e., IMQ NPs and CS-IMQ NPs, exhibited a cytotoxic effect and reduced cell viabilities in all cervical cancer cells compared to free IMQ (Figure 4A–C and Supplementary Tables S1–S4). Moreover, the cytotoxic effect was more pronounced in the higher concentrations used (25, 50, and 100 µg/mL). IMQ NPs, however, showed more cytotoxic effects compared to CS-IMQ NPs, which might be due to the faster release of the drug exhibited by IMQ NPs compared to their CS-coated counterparts (Supplementary Tables S1–S4). It seems that the most responsive cells by order were HeLa, C-33 A, and Ca-Ski, with cell viabilities of 38.9%, 41.1%, and 43.6 at 100 µg/mL, respectively, when treated with IMQ NPs (Figure 4A–C). Although there was a reduction in the viability in VEK/E6 E7, all cells maintained a minimum viability of about 60% even with higher concentrations of IMQ NPs (Figure 4D). 

### 2.7. Detection of the Inflammatory Potential of the IMQ NPs

**Figure 5:**
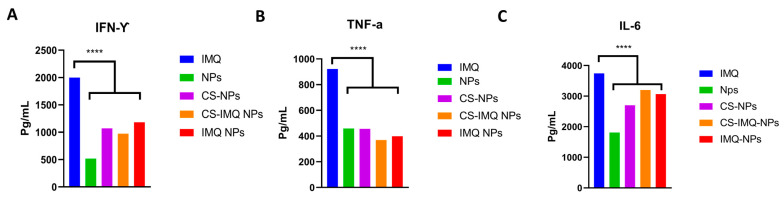
The induction of inflammatory cytokines (**A**) IFN-γ, (**B**) TNF-α, (**C**) IL-6 in VEK/E6 E7 in vaginal epithelium after the treatment with free IMQ and IMQ-loaded NPs. Drug-free NPs were used as control. Data are represented as mean ± SD (*n* = 3). Statistical significance was obtained with *p*-values ≤ 0.05, and **** *p* < 0.0001.

Imiquimod has been associated with several side effects, which might lead to the discontinuation of treatment. Topical side effects includes redness, itching, burning, and swelling at the site of administration [4,36]. Some patients also exhibited some flu-like symptoms such as fever, chills, headache, muscular pains, and weariness [4,36]. IMQ gastrointestinal side effects can include nausea, vomiting, and diarrhea [4,36]. These side effects might be caused by the stimulation of inflammatory cytokines mention above [35]. Although stimulating the innate immune system is the main anticancer mechanism of IMQ, an overdelivery of drugs to the site of action might unnecessarily exacerbate side effects. Thus, the utilization of nanoparticles can deliver the sufficient amount of drugs and reduce side effects [22]. One of our goals was to enhance the cytotoxic effect in CC cells but have minimal side effects at the intended site of application, which is vaginal tissues. In VEK/E6 E7, our results indicate that IMQ NPs and CS-IMQ NPs stimulate IFN-γ, TNF-α, and IL-6 to lower extents, which were significantly less than free IMQ (≈50% less with TNF-α and IFN-γ, and 18% less with IL-6) (Figure 5A–C), which might indicate that IMQ-loaded NPs are associated with fewer local side effects. 

## 3. Conclusions

In conclusion, our results show that our new delivery system of IMQ NPs either CS-uncoated or -coated loaded in an situ gel has good loading capacity, extended drug release, drug penetration, viscosity, and gelation temperature suitable for vaginal application. Moreover, this system released bioactive IMQ, which reduced the viability of cervical cancer cells and had a better safety profile on normal vaginal epithelium. Although further in vivo investigation is needed, these formulations offer promising results for a safer and more effective local treatment of cervical cancer. 

## 4. Materials and Methods

### 4.1. Materials 

Imiquimod (IMQ), poly (D, L-lactide-co-glycolide) (50:50) (Mw: 12,000 Da (PLGA), polycaprolactone (PCL, Mw; 42,000 Da), poly (lactide-co-caprolactone) (PLA-PCL) (lactide–caprolactone 82:14), poly (L-lactide-co-caprolactone-co-glycolide) (PLGA-PCL) (L-lactide–glycolide–caprolactone 70:10:20 molar ratio) were purchased from Sigma-Aldrich Chemical Co. (St. Louis, MO, USA). Polyvinyl alcohol (PVA) with an Mw of 16,000 Da and dichloromethane (DCM) were obtained from Acros Organics (Geel, Belgium). All other reagents and chemicals were of analytical grade. 

### 4.2. Preparation of IMQ NPs and CS-IMQ NPs

IMQ NPs were prepared using the emulsification–solvent evaporation technique, as described by [27]. Several polymers (PLGA, PCL, PLA-PCL, and PLGA-PCL) were employed to fabricate IMQ NPs. To create the IMQ NPs, an organic phase was prepared by dissolving 10 mg of IMQ and 50 mg of polymer in 10 mL of dichloromethane at room temperature. The organic solution was then added to a 20 mL aqueous phase containing a 1% polyvinyl alcohol (PVA) solution. The mixture was emulsified using a probe sonicator for 5 min on the ice at 60% amplitude to create an emulsion. The emulsion was further stirred for 4 h at room temperature to allow solvent evaporation. The resulting IMQ NPs were collected by centrifugation at 30,000 rpm for 30 min to eliminate the PVA solution and unloaded IMQ. The IMQ NP pellets were subsequently resuspended in 10 mL of distilled water.

To create positively charged nanoparticles, the surface of the IMQ NPs was coated with CS [37].The pellets of IMQ NPs were mixed with 10 mL of CS solution (0.5 mg/mL; 0.5% *w*/*v* acetic acid) and stirred for 3 h to produce CS-IMQ NPs. Then, IMQ NPs and CS-IMQ NPs were freeze-dried at −60 °C and 0.01 mbar for 72 h in a laboratory-scale freeze dryer (Alpha 1–4 LD Plus, Martin Christ Gefriertrocknungsanlagen GmbH, Osterode am Harz, Germany) and stored at 4 °C. The formulation process was conducted in triplicate to ensure reproducibility. Table 5 represents the compositions of the different formulations of IMQ-loaded NPs. 

### 4.3. Particle Size Distribution and Zeta Potential Measurement

The particle size and polydispersity index (PDI) were performed using a Zetasizer Nano ZS instrument (Malvern Instruments, Malver, UK) using the dynamic light scattering (DLS) mode. The dispersions of IMQ NPs and CS-IMQ NPs were appropriately diluted to a 1:100 ratio at 25 °C. Furthermore, the zeta potential of both IMQ NPs and CS-IMQ NPs was measured at 25 °C using the Laser Doppler Velocimetry (LDV) mode with the same Zetasizer Nano ZS instrument. Each experiment was repeated three times.

### 4.4. Encapsulation Efficiency and Loading Capacity Determination

An indirect method was used to determine the encapsulation efficiency (EE) and loading capacity (LC) of IMQ within the formulations in the supernatant during preparation. Freshly prepared IMQ NP and CS-IMQ NP dispersions were centrifuged at 30,000 rpm. 

The amount of free IMQ was analyzed in the supernatant using a modified reversed-phase HPLC method based on that described by [38]. The HPLC system used in this study consisted of a Waters^TM^ 600 controller, USA, equipped with a Waters^TM^ 2487 Dual λ Absorbance detector, a Waters^TM^ 1252 Binary pump, and a Waters^TM^ 717 Plus Autosampler, all controlled by Empower^®^ 3 Built 3471 Waters software. For the HPLC analysis, a mobile phase consisting of acetonitrile–acetate buffer (pH 4.0; 100 mM)–diethylamine (30:70:0.15 *v*/*v*) was used. The mobile phase flowed through a reversed-phase C_18_ column (µ-Bondapak^TM^, 4.6 × 150 mm, 10 µm particle size) at a rate of 1 mL/min. Each IMQ sample was injected into the HPLC system with an injection volume of 20 μL and detected by the UV detector at 242 nm.

EE% was calculated according to Equation (1):

(1) $$\mathrm{EE}\%=\frac{{\mathrm{IMQ}}_{\mathrm{total}}-{\mathrm{IMQ}}_{\mathrm{free}}}{{\mathrm{IMQ}}_{\mathrm{toal}}}\times 100$$

IMQ_total_ is the amount of drug added, while IMQ_free_ is the free amount of drug in the supernatant solutions. 

The LC% was calculated by dividing the encapsulated amount of IMQ by the total weight of NPs after lyophilization, according to Equation (2):

(2) $$\mathrm{EE}\%=\frac{\mathrm{The}\;\mathrm{encapsulated}\;\mathrm{amount}\;\mathrm{of}\;\mathrm{IMQ}}{\mathrm{Total}\;\mathrm{of}\;\mathrm{NPs}}\times 100$$

### 4.5. In Vitro Release Study

The in vitro release profile of IMQ from NPs and CS NPs was evaluated using the dialysis bag method. Dialysis bags with a molecular weight cutoff of 8–14 kDa (Livingstone, NSW, Australia) were soaked in distilled water for 12 h before use. The lyophilized formulations were dispersed in an appropriate citrate buffer (pH 4.5) volume (equivalent to 1 mg of IMQ) and sealed inside the dialysis bags. These bags were then immersed in beakers containing acetate buffer (50 mL). To maintain sink conditions, 0.2% *w*/*v* Tween 80 was added to the buffer solution. The beakers were placed in a thermostatic shaker set at 37 °C and 100 rpm. A lot of previous studies used a thermostatic shaker for determining drug release for vagina delivery [33,38]. At predetermined time intervals, 2 mL samples were withdrawn from the release medium at 0.25, 0.5, 1, 2, 4, 8, 12, 24, and 48 h. An equal volume of fresh buffer was added to maintain sink conditions. The collected samples were filtered using a 0.45 μm syringe filter and then analyzed by HPLC to quantify the amount of released IMQ. An IMQ suspension was used as a control. All experiments were conducted in triplicate to ensure reproducibility.

To understand the kinetics of the drug release mechanism, the release data were fitted to various kinetic models, including the zero-order model (cumulative% release vs. time), first-order model (log% drug remaining vs. time), Higuchi’s model (cumulative% drug release vs. square root of time), and Korsmeyer–Peppas plot (log of cumulative drug release vs. log time). The R^2^ values were computed to determine the goodness of fit for the linear regression curves obtained from the models.

### 4.6. The Particle Morphology 

The surface morphology of the selected freeze-dried IMQ NPs and CS-IMQ NPs was examined using a scanning electron microscope (JSM-6360 LV, JEOL, Tokyo, Japan). The freeze-dried samples were fixed onto carbon tape and coated with a thin layer of gold in a high-vacuum evaporator (JFC-1100 fine coat ion sputter; JEOL) under an argon atmosphere to enhance the conductivity of the samples and improve imaging quality. The coated samples were then scanned using the scanning electron microscope at an acceleration voltage of 10 kV, and photomicrographs were captured to visualize the surface characteristics and morphology of the particles.

### 4.7. Preparation of Thermosensitive In Situ Hydrogels

In situ hydrogels were prepared using the Cold technique, according to research performed by Matthew et al. in 2002 [39]. In brief, poloxamer 407 (20% *w*/*v*) and poloxamer 188 (2.5% *w*/*v*) were accurately weighed and dissolved in a 0.9% NaCl solution in a vial. The vials were added into the refrigerator overnight to cool and store at 4 °C. Thermosensitive in situ hydrogels were mixed with lyophilized IMQ NPs or CS-IMQ NPs after reconstitution with distilled water in an ice bath with a magnetic stirrer. The NPs were evenly distributed throughout the gel and reached a uniform and homogenous texture. Furthermore, free IMQ (1 mg/mL)-loaded in situ hydrogel was prepared using the same method and served as a control.

### 4.8. Characterization of Thermosensitive In Situ Hydrogels

The developed thermosensitive in situ gel formulations were visually inspected in the light against both white and black backgrounds to determine their clarity. The goal of this examination was to compare the formulations’ clarity before and after gelation. To determine the pH of in situ hydrogels at room temperature, a calibrated digital pH meter was used. To ensure the accuracy and dependability of the findings, the pH measurements were carried out in triplicate.

### 4.9. Determination of the Sol–Gel Transition Temperature

The tube inversion technique was used with a magnetic bar, and 2 g of formulation was incubated in an ice water bath for at least 15 min to allow the samples to thermally acclimate to the bath temperature. From 10 to 40 °C, the bath temperature was then raised gradually (1 °C min^−1^) while being stirred at 300 rpm. The gelation temperature is the temperature at which the magnetic stir bar completely stops rotating. The gelation time was measured after at least 15 min of incubation of polymer solutions in an ice water bath. Once the magnetic stir bar had stopped rotating, the formulations were heated to a temperature of 37 °C while being stirred at 300 rpm. During this time, gelation was noted. This period was recorded as the gelation time. All assays were performed in triplicate.

### 4.10. Viscosity Measurement

The viscosity of thermosensitive in situ hydrogel NPs was measured using a Sine-wave Vibro viscometer, SV-10 Series, manufactured by A&D Company, Ltd. (Tokyo, Japan). Throughout the duration of the viscosity measurement process, a shear rate of 100 s^−1^ was maintained. The measurements were conducted at 25 °C and 34 °C to investigate the thermo-gelling behavior of the ISG.

### 4.11. Ex Vivo Permeation of IMQ through the Vaginal Mucosa

PLGA-PCL (F4) was chosen for the ex vitro study based on the particle size, production yield, and EE%. Using Franz diffusion cells and sheep vaginal tissue, the incorporated IMQ NPs or CS-IMQ NPs with thermosensitive in situ hydrogels were tested for ex vivo permeability. Due to their resemblance to the female cervix and vagina, sheep’s cervix and vagina were deemed suitable for testing vaginal delivery systems. These tissues can also be easily obtained, prepared, and examined in a controlled laboratory environment [40]. They were placed in a cold Krebs buffer with a pH of 7.4 to guarantee the preservation of tissue viability throughout transport. After that, the healthy vaginal mucosa was separated from the adjacent tissue using surgical scissors and a scalpel. 

A diffusional area (1.67 cm^2^) of vaginal mucosa was mounted between the donor and receptor chambers of a Franz diffusion cell. Citrate-phosphate buffer (pH 4.5; 12 mL) was added to the receptor chambers, which were then thermostatically heated to 37 °C and magnetically stirred at 500 rpm to ensure that the receptor solution was thoroughly mixed. One mL of the F4 and CS-F4 was placed in the donor chambers. To measure the permeability of IMQ through the vaginal mucosa, samples of the receptor fluid were taken at intervals of 1, 2, 3, 4, 5, and 8 h. Fresh receptor solution was added to the receptor chamber following each sampling. The amounts of IMQ in the receptor fluid were determined using HPLC.

### 4.12. In Vitro Cytotoxicity Studies

The cytotoxicities of IMQ NPs (F4), CS-IMQ NPs (CS-F4), and the controls were evaluated in normal human vaginal cells (VK/E6E7) (ATCC^®^ CRL-2616™), cervical adenocarcinoma cells (HeLa) (ATCC^®^CRM-CCL-2™), metastatic cervical carcinoma HPV positive cells (Ca-Ski) (ATCC^®^CRL-1550™), and cervical carcinoma cells (C 33-A) (ATCC^®^HTB-31^™^). All cells were obtained from American type culture collection (ATCC, Manassas, VA, USA). Cells were maintained at 37 °C, and 5% CO_2_, and were cultured based on ATCC recommendations. To perform the cytotoxicity assay, cells were seeded in a 96-well culture plate at a density of 5000 cells/well in 100 μL culture media and incubated for 24 h. Cells were then treated with a serial dilution of the IMQ-loaded nanoparticles, free IMQ, or NPs only. After incubation at 37 °C for 72 h, media were replaced by 100 µL/well of MTT solution, 0.5 mg/mL, in PBS and incubated at 37 °C for another 3 h. The MTT solution was removed and the purple formazan crystals formed at the bottom of the wells were dissolved using DMSO. Then, absorbance was measured at 540 nm using a Spectramax 250 microplate reader (Molecular device, San Jose, CA, USA). Cell viability (%) was calculated as [optical density (OD) of treated cells/OD of control cells] × 100 [41].

### 4.13. Detection of the Inflammatory Potential of the IMQ NPs

Human inflammatory cytokines known to be stimulated by IMQ (IL6, IFN-gamma, and TNF-alpha) were measured in normal vaginal epithelium VEK/E6E7 [35] after treating the cells with IMQ-loaded NPs, and the free drug following manufacturer protocols (Cytokine ELISA Kits, ENZO life science, Farmingdale, NY, USA).

### 4.14. Statistical Data Analysis

GraphPad Prism 8.0.1 software (GraphPad Software Inc., San Diego, CA, USA) was used for statistical analysis and graph plotting. Unless otherwise specified, all tests were carried out in triplicate. The results are shown as mean SED, with *p* > 0.05 deemed significant. (*n* = 3 independent samples).

## Figures and Tables

**Table 1:** The physicochemical characterization of IMQ NPs and CS NPs.

Polymeric Nanoparticles	Particle Size (nm)	PDI	Zeta Potential (mV)
PLGA	216.4 ± 8.1	0.207 ± 0.017	−20.13 ± 2.27
PCL	189.1 ± 3.9	0.243 ± 0.015	−13.63 ± 1.62
PLA-PCL	237.3 ± 4.7	0.201 ± 0.021	−18.67 ± 1.45
PLGA-PCL	206.7 ± 8.7	0.195 ± 0.007	−16.51 ± 1.37
CS-PLGA	225.9 ± 7.5	0.277 ± 0.061	23.23 ± 3.11
CS-PCL	232.4 ± 3.9	0.286 ± 0.048	17.03 ± 1.51
CS-PLA-PCL	278.2 ± 5.4	0.229 ± 0.023	20.46 ± 2.08
CS-PLGA-PCL	218.5 ± 9.6	0.322 ± 0.037	18.01 ± 1.08

**Table 2:** The yield%, encapsulation efficiency (EE%), and loading capacity (LC%) of IMQ NPs and CS-IMQ NPs (mean ± SD, *n* = 3).

Polymeric Nanoparticles	Yield%	EE%	LC%
PLGA	76.43	51.09 ± 4.61	8.14 ± 0.29
PCL	73.21	46.81 ± 1.78	7.18 ± 0.43
PLA-PCL	87.22	39.86 ± 2.46	6.37 ± 0.62
PLGA-PCL	85.76	61.48 ± 5.19	10.32 ± 0.85
CS-PLGA	81.15	45.71 ± 2.84	6.15 ± 0.35
CS-PCL	88.62	38.74 ± 1.65	5.41 ± 0.79
CS-PLA-PCL	84.63	37.73 ± 2.88	5.81 ± 0.65
CS-PLGA-PCL	86.32	49.26 ± 2.73	7.67 ± 0.24

**Table 3:** In vitro release kinetics models of IMQ-loaded NPs and CS NPs.

Correlation Coefficient (R^2^)					
Formulations	Zero-Order	First-Order	Higuchi’s Model	Korsmeyer–Peppas Model	
	R^2^				*n*
F1	0.768	0.972	0.995	0.997	0.532
F2	0.611	0.739	0.976	0.993	0.426
F3	0533	0.674	0.941	0.989	0.431
F4	0.905	0.987	0.982	0.993	0.360
CS-F1	0.718	0.934	0.951	0.996	0.382
CS-F2	0.814	0.944	0.973	0.991	0.422
CS-F3	0.901	0.953	0.974	0.998	0.361
CS-F4	0.934	0.902	0.983	0.993	0.387

**Table 4:** Vaginal permeation parameters of formulations through the sheep vaginal mucosa (*n* = 3, mean ± standard deviation).

Formulations	Js (μg/cm^2^·h)	P (cm/h × 10^−3^)	ER
Control	0.198 ± 0.054	0.397 ± 0.006	-
F4	4.263 ± 0.872 *	8.526 ± 0.756 *	21.5
CF4	5.905 ± 0.711 *	11.810 ± 2.006 *	29.8

The data are presented as mean ± standard deviation. The statistical analysis was performed using one-way ANOVA with Tukey’s post-test to compare the treatments, and each parameter was analyzed separately. Statistical significance was considered at *p* < 0.05. The asterisk (*) denotes statistically significant differences compared to the control group. The parameters evaluated in this study include steady-state flux (Js), permeability coefficient (P), and enhancement ratio (ER).

**Table 5:** The composition of IMQ NPs and CS-coated NPs.

Codes Ingredients	PLGA (mg)	PCL (mg)	PLA-PCL (mg)	PLGA-PCL (mg)	CS (mg/mL)	IMQ (mg)
F1	50	-	-	-	-	10
F2	-	50	-	-	-	10
F3	-	-	50	-	-	10
F4	-	-	-	50	-	10
CS-F1	50	-	-	-	0.5	10
CS-F2	-	50	-	-	0.5	10
CS-F3	-	-	50	-	0.5	10
CS-F4	-	-	-	50	0.5	10

PLGA: poly (lactide-co-glycolide); PCL: poly (caprolactone); PLA-PCL: poly(L-lactide-co-caprolactone); PLGA-PCL: poly(L-lactide-co-caprolactone-co-glycolide); CS: chitosan; IMQ: imiquimod.

## Data Availability

All data are available in the main manuscript and supplementary data.

## Supplementary Materials

The following supporting information can be downloaded at: https://www.mdpi.com/article/10.3390/gels9090713/s1. Table S1: Statistical significant difference of cytotoxicity between different treatment groups in Ca-Ski cells, Table S2: Statistical significant difference of cytotoxicity between different treatment groups in HeLa cells, Table S3: Statistical significant difference of cytotoxicity between different treatment groups in C-33 A cells, Table S4: Statistical significant difference of cytotoxicity between different treatment groups in VK/E6E7 cells.
