# Supplementary material for: Imiquimod-Loaded Chitosan-Decorated Di-Block and Tri-Block Polymeric Nanoparticles Loaded In Situ Gel for the Management of Cervical Cancer
Source: Gels. 2023 Sep 3;9(9):713. doi: 10.3390/gels9090713 (PMC10530705; doi:10.3390/gels9090713)
Supplement: Supplementary file 1 [file gels-09-00713-s001.zip › gels-2550973-supplementary.pdf]

Table S1. Statistical significance between different treatment groups in Ca-Ski cells

NPs Conc. (µg/mL)	**0.78**	**1.56**	**3.125**	**6.25**	**12.5**	**25**	**50**	**100**
IMQ vs. CS-IMQ NPs	ns	**	ns	****	****	****	****	****
**IMQ vs. IMQ NPs**	ns	***	ns	**	****	****	****	****
IMQ vs. CS-NPs	ns	ns	****	****	****	****	****	****
IMQ vs. NPs	ns	ns	****	****	****	****	****	****
**CS-IMQ NPs vs. IMQ NPs**	ns	ns	ns	***	****	****	****	****
CS-IMQ NPs vs. CS-NPs	ns	***	****	****	****	****	****	****
CS-IMQ NPs vs. NPs	ns	**	****	****	****	****	****	****
**IMQ NPs vs. CS-NPs**	*	****	****	****	****	****	****	****
**IMQ NPs vs. NPs**	*	****	****	****	****	****	****	****
CS-NPs vs. NPs	ns	ns	ns	ns	***	*	***	****

p-values of < 0.05 were considered statistically significance, where * *p< 0.05,* ** *p< 0.005,* *** *p* < 0.001, and **** *p* < 0.0001

Table S2. Statistical significance between different treatment groups in HeLa cells

NPs Conc. (µg/mL)	**0.78**	**1.56**	**3.125**	**6.25**	**12.5**	**25**	**50**	**100**
IMQ vs. CS-IMQ NPs	ns	***	ns	ns	***	ns	ns	ns
**IMQ vs. IMQ NPs**	ns	****	****	****	****	****	****	****
IMQ vs. CS-NPs	ns	**	****	****	****	****	****	****
IMQ vs. NPs	ns	***	****	****	****	****	****	****
**CS-IMQ NPs vs. IMQ NPs**	ns	**	****	****	****	****	****	****
CS-IMQ NPs vs. CS-NPs	ns	****	****	****	****	****	****	****
CS-IMQ NPs vs. NPs	ns	****	****	****	****	****	****	****
**IMQ NPs vs. CS-NPs**	ns	****	****	****	****	****	****	****
**IMQ NPs vs. NPs**	ns	****	****	****	****	****	****	****
CS-NPs vs. NPs	ns	ns	ns	ns	ns	ns	ns	ns

p-values of < 0.05 were considered statistically significance, where * *p< 0.05,* ** *p< 0.005,* *** *p* < 0.001, and **** *p* < 0.0001

Table S3. Statistical significance between different treatment groups in C-33 A cells

NPs Conc. (µg/mL)	**0.78**	**1.56**	**3.125**	**6.25**	**12.5**	**25**	**50**	**100**
IMQ vs. CS-IMQ NPs	ns	ns	ns	****	****	****	ns	*
**IMQ vs. IMQ NPs**	ns	*	****	****	****	****	****	****
IMQ vs. CS-NPs	ns	ns	****	****	****	****	****	****
IMQ vs. NPs	ns	ns	****	****	****	****	****	****
**CS-IMQ NPs vs. IMQ NPs**	ns	*	****	****	****	****	****	****
CS-IMQ NPs vs. CS-NPs	ns	ns	****	****	****	****	****	****
CS-IMQ NPs vs. NPs	ns	ns	****	****	****	****	****	****
**IMQ NPs vs. CS-NPs**	ns	**	****	****	****	****	****	****
**IMQ NPs vs. NPs**	ns	**	****	****	****	****	****	****
CS-NPs vs. NPs	ns	ns	ns	ns	ns	ns	ns	ns

p-values of < 0.05 were considered statistically significance, where * $p< 0.05$, ** $p< 0.005$, *** $p < 0.001$, and **** $p < 0.0001$

Table S4. Statistical significance between different treatment groups in VK/E6E7 cells

NPs Conc. (µg/mL)	**0.78**	**1.56**	**3.125**	**6.25**	**12.5**	**25**	**50**	**100**
IMQ vs. CS-IMQ NPs	ns	*	ns	ns	*	**	ns	ns
**IMQ vs. IMQ NPs**	ns	ns	****	****	****	****	****	****
IMQ vs. CS-NPs	ns	****	****	****	****	****	****	****
IMQ vs. NPs	ns	****	****	****	****	****	****	****
**CS-IMQ NPs vs. IMQ NPs**	ns	ns	****	****	****	****	****	****
CS-IMQ NPs vs. CS-NPs	ns	****	****	****	****	****	****	****
CS-IMQ NPs vs. NPs	ns	****	****	****	****	****	****	****
**IMQ NPs vs. CS-NPs**	ns	****	****	****	****	****	****	****
**IMQ NPs vs. NPs**	ns	****	****	****	****	****	****	****
CS-NPs vs. NPs	ns	ns	ns	ns	***	**	ns	ns

p-values of < 0.05 were considered statistically significance, where * $p< 0.05$, ** $p< 0.005$, *** $p < 0.001$, and **** $p < 0.0001$
